# Evaluation of the Hyaluronic Acid Versus the Injectable Platelet-Rich Fibrin in the Management of the Thin Gingival Phenotype: A Split-Mouth Randomized Controlled Clinical Trial

**DOI:** 10.7759/cureus.25104

**Published:** 2022-05-18

**Authors:** Nai H Faour, Suleiman Dayoub, Mohammad Y Hajeer

**Affiliations:** 1 Department of Periodontology, Damascus University Faculty of Dentistry, Damascus, SYR; 2 Department of Orthodontics, University of Damascus Faculty of Dentistry, Damascus, SYR

**Keywords:** probing depth, bleeding on probing, gingival index, i-prf, injectable platelet-rich-plasma, hyaluronic acid, gingival thickness, phenotype, gingiva

## Abstract

Background

Several procedures have been used to enhance thin gingival phenotype and the majority of these procedures have been surgical. A new minimally invasive approach that involved multiple injections of platelet-rich fibrin (i-PRF) to enhance the thin gingival phenotype has been proposed. As the hyaluronic acid (HA) and the i-PRF share similar properties in terms of promoting periodontal regeneration, the present trial aimed to evaluate the effectiveness of multiple injections of the i-PRF in patients with thin gingival phenotypes in comparison with those of the HA in increasing the gingival thickness (GT) and the keratinized tissue width (KTW).

Materials and methods

Eighty-four sites from 14 systematically healthy patients who had thin gingival phenotypes (GT ≤1 mm) were included in this split-mouth randomized controlled trial. For each patient, each side of the anterior mandible was randomly allocated to one of the two materials (HA or i-PRF). In the HA group, the selected sites of the gingiva were injected with cross-linked HA using a 30-gauge microneedle. In the i-PRF group, the i-PRF was injected in the same manner. This procedure was repeated in both groups three times with intervals of 7 days. The GT, KTW, and periodontal indices: gingival index (GI), bleeding on probing (BOP), and probing depth (PD) were measured at baseline, 1 month, and 3 months following the initial injection.

Results

The GT increased significantly in both groups at the three assessment times (p<0.001). The KTW also showed a statistically significant increase in the intragroup comparisons in both groups (p<0.05). No statistically significant difference was observed between the two groups at the three assessment times for the GT and the KTW (p>0.05).

The GI significantly decreased after 1 month and 3 months compared to the baseline values in both groups (p<0.05). The intergroup comparisons for the GI revealed no statistically significant differences at the three assessment times (p>0.05). As for the BOP and the PD, no statistically significant differences were found between the three assessment times (p>0.05) and between the two groups at each assessment time (p>0.05).

Conclusion

Multiple injections of the i-PRF and the HA in the thin gingival phenotype resulted in an increased GT and increased KTW, with no statistically significant differences between the two methods. Both minimally invasive techniques were more effective in improving the GT rather than the KTW.

## Introduction

Recently, studies focusing on the morphology and dimensions of the periodontal soft and hard tissues have increased significantly in the field of periodontics. In daily clinical practice, identifying the different phenotypes of the gingiva before dental procedures has a significant impact on the treatment plan, functional and esthetic outcome, and prognosis of restorative and regenerative therapies [1].

Characteristics of the gingiva and underlying alveolar bone vary among individuals, and within the same individual in different parts of the oral cavity, and are affected by multiple factors such as; events that occur during tooth eruption, alveolar bone dimensions, and tooth morphology [2,3].

Seibert and Lindhe suggested the term “gingival biotype” which is defined as the thickness of the gingiva in the facio-palatal direction. They categorized the gingiva into “thick-flat” and “thin-scalloped”. Thick gingiva is usually associated with wide zones of keratinized tissue and flat gingival contour, whereas thin gingiva is associated with a narrow band of keratinized tissue, and a scalloped gingival margin [4]. Later, the World Workshop of Periodontics in 2017, recommended using the term “phenotype” instead of “biotype” [5]. The phenotype of an organ is based on “genetic traits and environmental factors; its expression includes the ‘biotype’ which is only based on the genetic factors thus can’t be modified.” The periodontal phenotype as a definition is the combination of gingival phenotype and bone morphotype (buccal bone plate thickness) [5]. Gingival phenotype involves gingival thickness (GT) and keratinized tissue width (KTW).

While bone morphotype can only be assessed by cone-beam computed tomography system (CBCT), the gingival phenotype can be assessed in easier, more stable, and reproducible methods like transgingival probing, probe transparency, modified calipers, and ultrasonic devices [6].

The probe transparency method is considered both classification and assessment method for the gingival phenotype. The periodontal probe is inserted in the gingival sulcus, the gingival phenotype is deemed to be thin if the periodontal probe is visible through the gingival tissue (GT is smaller than or equal to 1 mm), while it is considered thick when the periodontal probe is not visible through the gingival tissue (GT is greater than 1 mm) [5]. Different gingival phenotypes behave differently under similar conditions and clinical interventions. Trauma and inflammation are likely to cause the gingival recession with thin phenotypes, whereas pocket formation is more frequently seen with thick phenotypes [7].

In mucogingival surgeries, flap thickness predicts the outcome’s success and better identifies the surgical technique to be used. A flap thickness of greater than 0.8 mm is associated with complete root coverage, whereas a thinner flap of less than 0.8 mm results in partial root coverage as can be seen in Miller’s class 1 or 2 recession defects [7]. The diversion in the behavior of different tissue phenotypes indicates that thick tissues withstand trauma and subsequent recession, thus facilitating manipulation of tissue, and providing predictable surgical outcomes [8].

Hyaluronic acid (HA), a naturally occurring polysaccharide, is considered an important component of the extracellular matrix in connective tissues of the human body [9]. HA participates in various physiologic and structural processes that preserve tissue integrity, such as cellular and extracellular interactions, modulation of the inflammatory process, interactions with growth factors, tissue healing, collagen synthesis, regulation of the osmotic pressure, and tissue lubrication [10].

The unique biological and physiochemical properties of HA make it an interesting biomaterial for medical, cosmetic, and pharmaceutical applications. HA has been widely used in the dental field, specially periodontology, due to its bacteriostatic, fungistatic, anti-inflammatory, anti-edematous, osteoinductive, and pro-angiogenetic properties [11]. HA’s role in tissue regeneration and wound healing has gained huge interest in recent studies. These studies have believed that HA accelerates the wound healing process, and promotes regeneration, as it maintains the viability of oral fibroblasts, increases their proliferative and migratory abilities, and enhances the expression of genes encoding type III collagen and transforming growth factor-β3 [12].

Autologous blood concentrates show favorable results in the application site due to the higher concentrations of growth factors they carry. Among various platelet concen­trates, platelet-rich fibrin (PRF) was one of the most commonly used platelets concentrates in dentistry. It played a role in carrying cells for the purpose of tissue regeneration, treatment of various types of periodontal defects, gingival recessions, palatal wound closure, and wound healing [13]. PRF is obtained in a gel form which sometimes limits its clinical application when there is the necessity for a liquid form conducive to being injected [14].

In 2014, a liquid form of PRF was developed, by changing the type of the tube, centrifugation time, and speed; specifically, the blood is centrifuged in plastic tubes at 700 rpm for 3 min [15]. Injectable platelet-rich fibrin (i-PRF) prepared according to the low-speed centrifugation concept can provide a significant advantage for the regeneration process due to a higher presence of regenerative cells such as white blood cells, leading to higher concentrations of growth factors. I-PRF also induces higher fibroblast migration and expression of platelet-derived growth factor (PDGF), transforming growth factor B (TGF-B), and collagen-1 [13].

Various methods have been used for phenotype modification [16]. PRF derivatives have provided beneficial outcomes in increasing the GT and KTW [17,18]. However, most of the approaches used for this purpose were surgical and invasive in nature [16]. Ozsagir et al. proposed a new minimally invasive approach that involved multiple injections of i-PRF in enhancing the thin gingival phenotype [19]. As the HA and the i-PRF share similar properties in terms of promoting periodontal regeneration, collagen synthesis, and wound healing, as well as the reported positive effect of HA on interdental-papilla augmentation, this study aimed to evaluate the effect of multiple injections of HA on thin gingival phenotype and to compare this effect with that resulting from multiple injections of the i-PRF [20].

## Materials and methods

Study design and registration

This study is an interventional, single-blinded, randomized split-mouth, controlled trial. It was approved by the Scientific Research Committee at the University of Damascus Dental School (UDDS-522-24082020/SRC-2793). The study protocol was registered in the International Standard Randomised Controlled Trial Number (ISRCTN) database (Reference number: ISRCTN10040718) and was funded by the University of Damascus Postgraduate Research Budget (Reference number: 80017289987DEN).

Sample size calculation

G*power software version 3.1.9.4 was used to calculate the sample size considering the following: The effect size of GT was 0.79 (standard deviation=0.63) according to a previous study, the power of 90%, an alpha level of 0.05, and two-sample t-test as the statistical test [21]. The analysis revealed that 29 sites were required for each group. This number was increased to 42 sites to compensate for any unexpected dropouts. Six sites at the anterior mandible (central incisors, lateral incisors, and canines) were included for each patient, as each tooth was considered one site.

Participants, settings, and eligibility criteria

**Figure 1:**
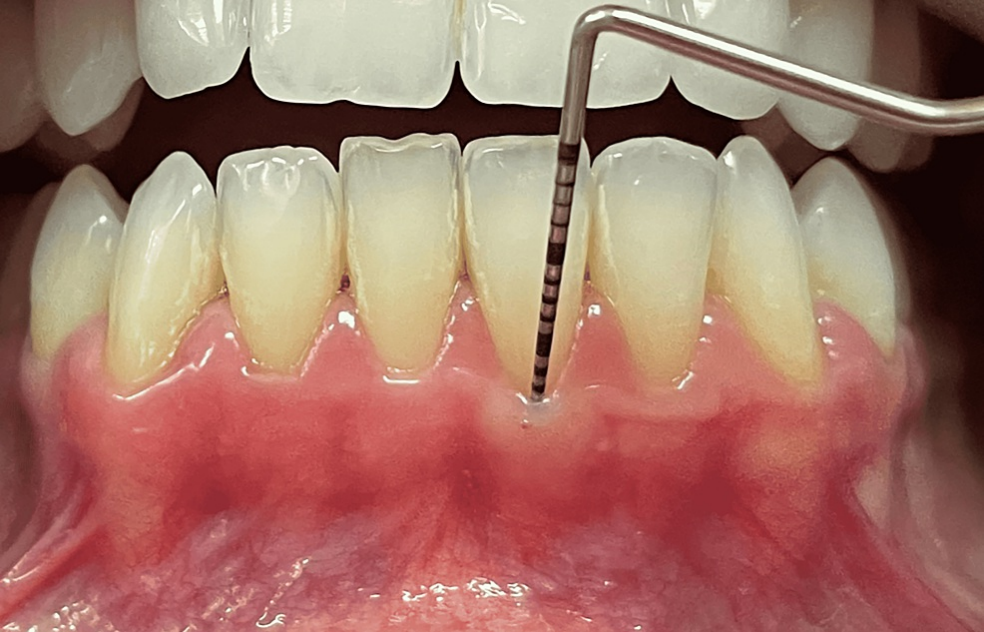
The probe can be visible through the gingival margin if the phenotype is thin.

Our study recruited 14 patients (84 sites) referred to the Department of Periodontology, Damascus University between April 2021 and August 2021. Patients eligible for this study were systematically healthy patients between 18 and 40 years old who had good oral hygiene and a thin gingival phenotype (GT ≤1 mm) in the lower anterior mandible, which was determined by the ‘probe transparency’ method. The gingival phenotype was considered thin if the periodontal probe inserted in the gingival sulcus was visible through the gingiva (GT ≤ 1 mm) (Figure 1). Patients were excluded if they were smokers, pregnant or lactating women, had chronic or aggressive periodontitis, had undergone previous periodontal surgery, patients with active orthodontic treatment, and patients who had blood anomalies, tooth mobility, bruxism, missing or supernumerary teeth. The procedure was fully explained to all patients, and informed approvals were obtained.

Randomization, allocation concealment, and blinding

Each side of the anterior mandible was randomly allocated to one of the two materials: A (HA-right side; i-PRF-left side) or B (HA-left side; i-PRF-right side). The allocation sequence was done using a computer random number generator (allocation ratio of 1:1). The allocation sequence was concealed in opaque sealed envelopes, which were identified with the initials of the patient’s name. For each patient, the envelope was opened immediately before the intervention. Patients were blinded during the intervention and follow-up sessions.

Interventional groups: group A: the hyaluronic acid group

**Figure 2:**
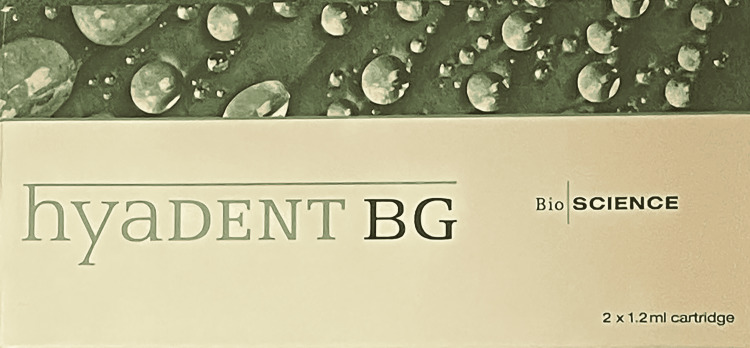
The hyaluronic acid used in the current trial.

**Figure 3:**
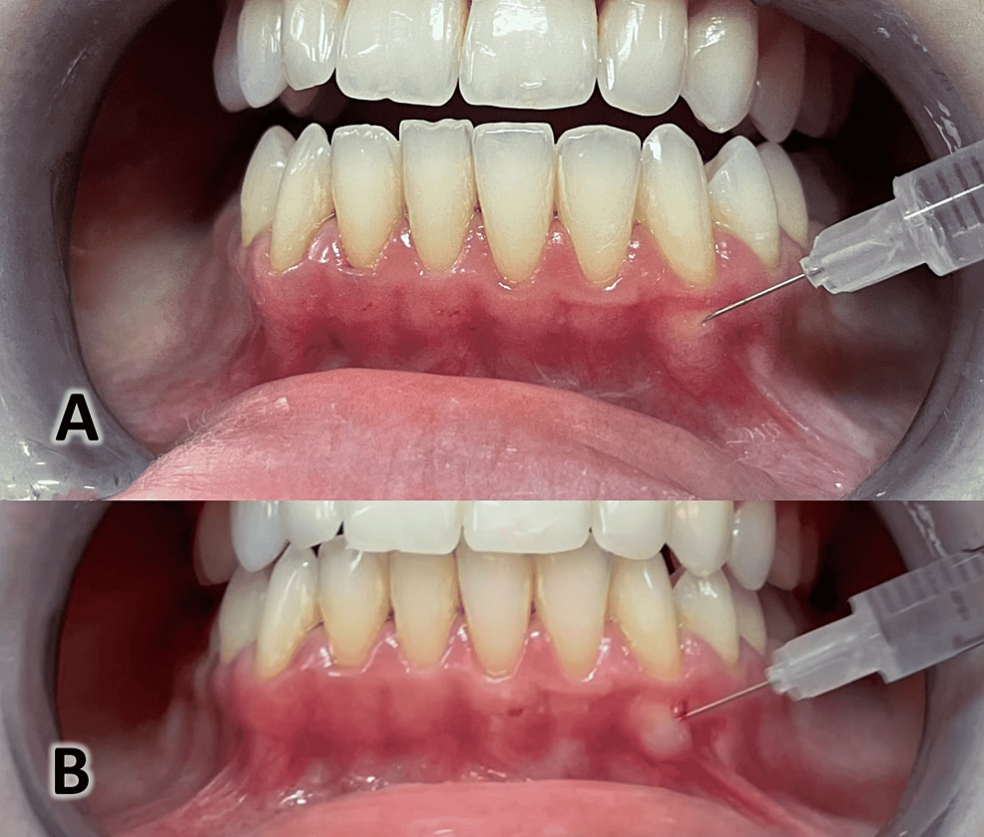
The injection of hyaluronic acid (HA). A) Injecting the HA until blanching of the gingiva is seen. B) The injection of the HA was apical to the mucogingival margin.

For this group, injectable HA gel (HyaDENT BG, BioScience GmbH, Germany; Figure 2) was used. Every 1 ml of this gel contains: 2 mg HA and 16 mg cross-linked HA. HA was transferred to a 1-ml microneedle (30 G × 8 mm needle, SHINA, insulin syringe needle) marked every 0.02 ml. Before the intervention and measuring the GT, the topical anesthetic spray was applied to the gingiva of the lower anterior region. Then, the selected sites of the gingiva were injected with HA on one side of the mandibular anterior region. Injecting HA was done at two points for each site in the attached gingiva at 3 mm apical to the free gingival margin in the facial side of the tooth until the blanching of the gingiva was seen and apical to the mucogingival junction (0.04 ml; Figure 3).

Group B: the i-PRF group

**Figure 4:**
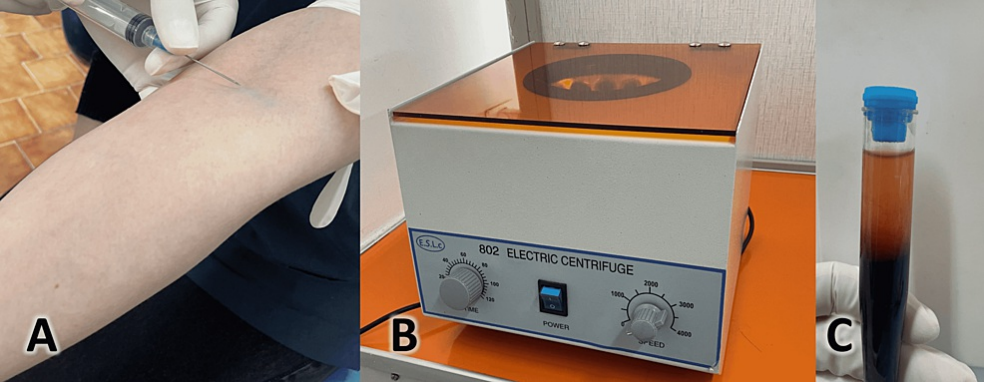
Preparation of the injectable platelet-rich fibrin (i-PRF). A) 5 ml of blood sample was collected just before the intervention. B) Blood was then placed in a plastic tube without any added material or coagulant and was centrifuged using the shown device at 700 rpm for 3 min. C) The tube containing the centrifugated blood.

**Figure 5:**
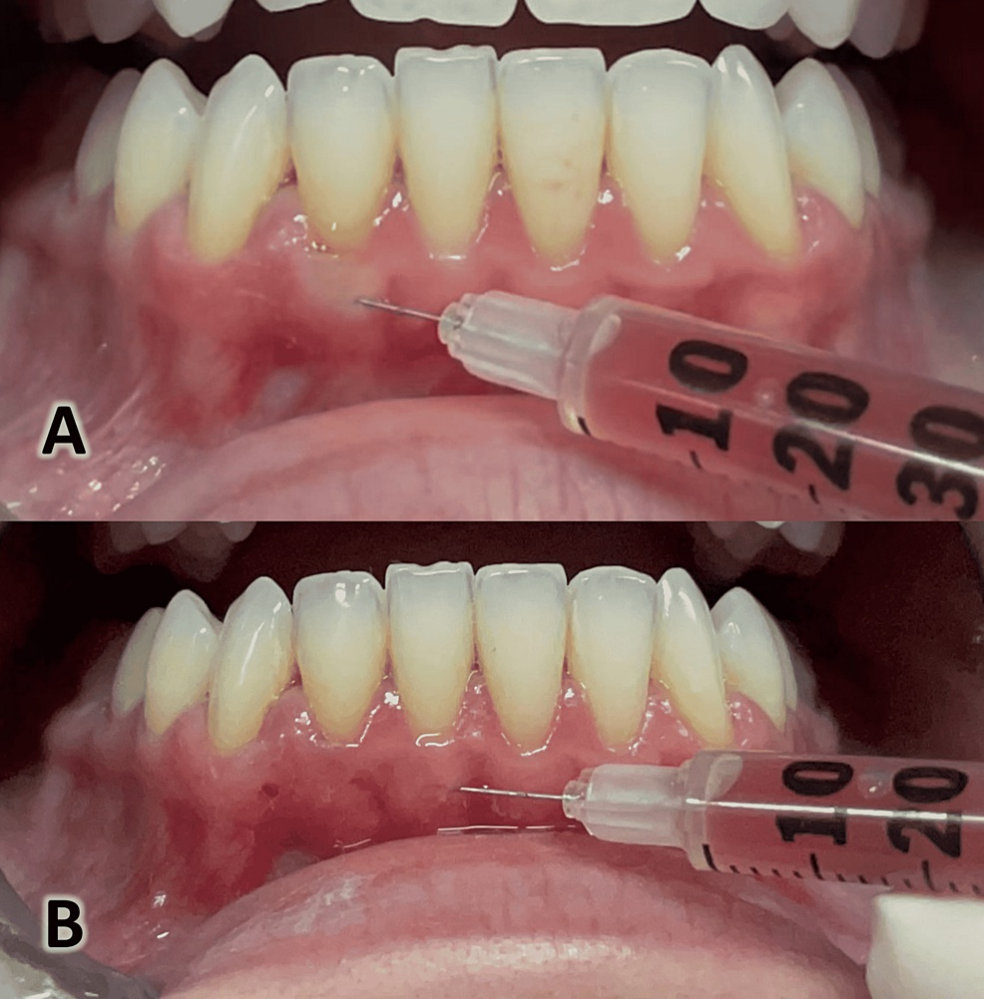
Injection of the platelet-rich fibrin (PRF). A) The injection is done until blanching of the gingiva is seen. B) The injection was positioned apical to the mucogingival margin.

I-PRF was prepared for each patient as follows: 5 ml of venous blood was collected from the patient in each session right before the intervention (Figure 4A). Blood was then placed in a plastic tube without any added material or coagulant; then, it was centrifuged using (E.S.L.c 802 electric centrifuge, ESSE3, Castelnuovo D.B, Italy (at 700 rpm for 3 min (Figures 4B-4C). I-PRF was also transferred to a marked 1 ml microneedle (30 G × 8 mm needle, SHINA™ insulin syringe needle, SHINA, Kongju-City, Korea). I-PRF was injected on the opposite side of the mandibular anterior region of the same patient, the same way used for injecting HA (Figure 5). The injecting procedure was repeated in both groups for three sessions, with 7 days between sessions. After the intervention, patients received oral hygiene instructions and were taught to use the roll technique when brushing the intervention area to minimize trauma.

Primary outcome measures: gingival thickness and keratinized gingival width

**Figure 6:**
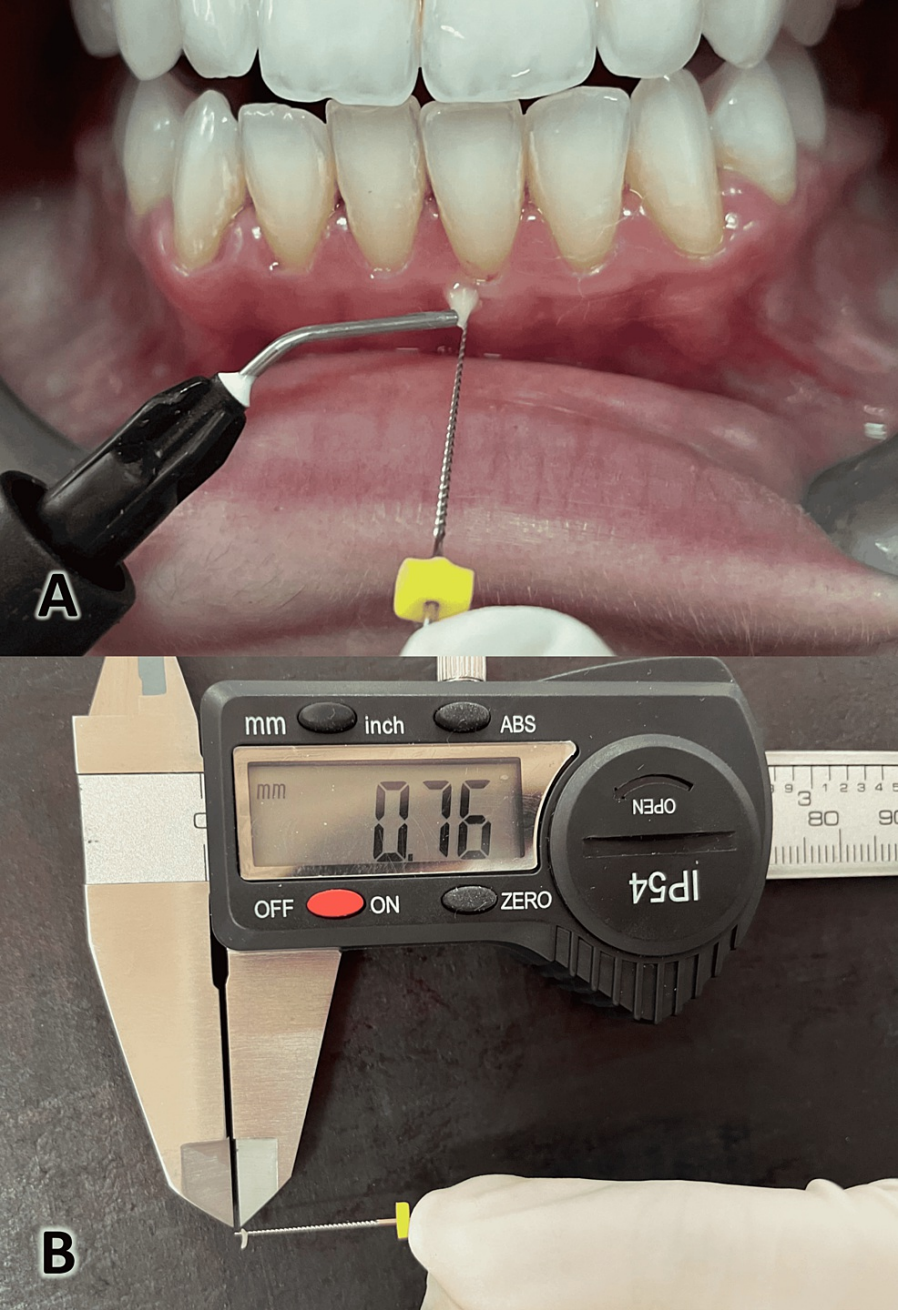
Measurement of the gingival thickness. A) A no. 15 endodontic file was inserted perpendicularly through the gingiva at 2 mm apical to the gingival margin through the soft tissue until a hard surface was reached. A flowable light-curing composite was used to mark the penetration depth on the file. B) A digital caliper was used to measure the penetration depth between the file’s tip and the light-cured composite.

Clinical measurements were taken at baseline, 1 month, and 3 months after the intervention during the follow-up period and were done by a single calibrated examiner (NF). GT was measured using a no. 15 endodontic file (the file was inserted perpendicularly through the gingiva, 2 mm apical to the gingival margin through the soft tissue until a hard surface was reached). The flowable light-curing composite was used to mark the penetration depth on the file (Figure 6A) [22]. Then, a digital caliper was used to measure the penetration depth between the file’s tip and the light-cured composite (Figure 6B). The KTW was calculated from the gingival margin to the mucogingival junctions with the help of a periodontal probe (UNC-15) with a silicone disc; then, the measurement was also done with the digital caliper.

Secondary outcome measures: gingival index, bleeding on probing, and probing depth

Secondary outcomes were the gingival index (GI), the probing depth (PD) measurement, and the bleeding on probing (BOP) index [23,24]. The GI is used to assess the gingival condition based on the following criteria: 0=normal gingiva, 1=mild inflammation (slight change in color and slight edema but no BOP), 2=moderate inflammation (redness, edema, and glazing, bleeding on probing), 3=severe inflammation (marked redness and edema, ulceration with tendency to spontaneous bleeding). The scores of the four areas of the tooth (buccal, mesial, distal, lingual) can be summed and divided by four to give the GI for the tooth. PD represents the sulcus or pocket depth measured from the gingival margin to the bottom of the sulcus or pocket. This index was calculated at four sites (buccal, mesial, distal, lingual) for each tooth using a UNC-15 periodontal probe [25]. After probing the gingival sulcus, BOP was assessed using the UNC-15 periodontal probe. A positive score was given if bleeding occurred within 10-15 seconds. The BOP value was calculated as the percentage of bleeding gingival units out of the total number of sites examined per individual.

Statistical analysis

Data analysis was carried out in SPSS Program (17.0 V for Windows, SPSS Inc., Chicago, IL, USA). The normality of the data distribution was evaluated with the Shapiro-Wilk test. All parameters were shown to be not normally distributed. Mann-Whitney U test was used for detecting differences between the two groups at each assessment time, whereas the Wilcoxon signed-rank matched pairs test was used to detect significant differences for intragroup changes over time (i.e., between assessment times).

## Results

Fourteen patients were recruited, and 84 sites were examined. All included sites entered the final data analysis without dropouts. The mean age of the included patients was 29.71 (standard deviation: 7.47 years). Females comprised 64.3% (n=9) of the sample (Table 1). The GT increased in both groups and the intragroup comparisons between assessment times revealed significant changes (p<0.001; Table 2). No statistically significant differences were observed between the two groups at the three assessment times (p>0.05; Table 3).

**Table 1:** Sample basic characteristics

		Number	Percentage
Gender	Male	5	35.7%
	Female	9	64.3%
Age		Mean	Standard deviation
		29.71	7.47

**Table 2:** Pairwise comparisons of changes in the gingival thickness between assessment times in each group i-PRF: injectable platelet-rich fibrin; HA: hyaluronic acid *Wilcoxon test was used for the intragroup comparison (when the same variable was checked across multiple time points in the intragroup comparisons).

Variables		Mean difference	95% CI of the difference (lower bound-upper bound)		P-value*
i-PRF group	Baseline-1 month	0.762	0.715	0.808	<0.001
		1.02	0.962	1.08	
	Baseline-3 months	0.762	0.715	0.808	<0.001
		1.05	0.995	1.12	
	1 month-3 months	1.02	0.962	1.08	<0.001
		1.05	0.995	1.12	
HA group	Baseline-1 month	0.753	0.709	0.796	<0.001
		1.057	0.996	1.11	
	Baseline-3 months	0.753	0.709	0.796	<0.001
		1.09	1.03	1.15	
	1 month-3 months	1.057	0.996	1.11	<0.001
		1.09	1.03	1.15	

**Table 3:** Comparison of the gingival thickness between the two groups at the three assessment times (n=42) GT: gingival thickness; i-PRF: injectable platelet-rich fibrin; HA: hyaluronic acid *Mann-Whitney U test was used for the intergroup comparison (i-PRF and HA).

	Groups	Mean	Minimum value	Maximum value	Standard deviation	P-value*
GT-baseline	i-PRF	0.76	0.44	0.99	0.15	0.664
	HA	0.75	0.40	0.97	0.14	
GT-1 month	i-PRF	1.03	0.55	1.32	0.20	0.552
	HA	1.06	0.59	1.44	0.19	
GT-3 months	i-PRF	1.06	0.59	1.34	0.20	0.460
	HA	1.09	0.62	1.47	0.19	

The KTW also increased significantly in both groups and the intragroup comparisons between assessment times showed significant changes (p<0.05; Table 4). No statistically significant difference was observed between the two groups at the three assessment times (p>0.05; Table 5). The GI significantly decreased after 1 month and 3 months compared to the baseline value in both groups (p<0.05; Table 6). The intergroup comparisons for the GI revealed no statistically significant differences at the three assessment times (p>0.05; Table 7). As for the PD, no statistically significant differences were found between the three assessment times (p>0.05; Table 8) and between the two groups at each assessment time (p>0.05; Table 9). Likewise, there were no significant differences between the three assessment time regarding the bleeding on the probing index (p>0.05; Tables 10) and between the two groups at the three assessment times (p>0.05; Table 11).

**Table 4:** Pairwise comparisons of changes in the keratinized tissue width between assessment times in each group i-PRF: injectable platelet-rich fibrin; HA: hyaluronic acid *Wilcoxon test was used for the intragroup comparison (when the same variable was checked across multiple time points in the intragroup comparisons).

Variables		Mean difference	95% CI of the difference (lower bound-upper bound)		P-value*
i-PRF group	Baseline-1 month	4.06	3.83	4.29	<0.001
		4.07	3.84	4.30	
	Baseline-3 months	4.06	3.83	4.29	<0.001
		4.07	3.84	4.31	
	1 month-3 months	4.07	3.84	4.30	0.003
		4.07	3.84	4.31	
HA group	Baseline - 1 month	4.05	3.84	4.27	<0.001
		4.08	3.87	4.29	
	Baseline-3 months	4.05	3.84	4.27	<0.001
		4.09	3.87	4.31	
	1 month-3 months	4.08	3.87	4.29	<0.001
		4.09	3.87	4.31	

**Table 5:** Comparison of the keratinized tissue width between the two groups at the three assessment times (n=42) KTW: keratinized tissue width; i-PRF: injectable platelet-rich fibrin; HA: hyaluronic acid *Mann-Whitney U test was used for the intergroup comparisons (i-PRF versus HA).

	Groups	Mean	Minimum value	Maximum value	Standard deviation	P-value*
KTW-baseline	i-PRF	4.07	2.59	5.80	0.75	0.911
	HA	4.06	2.80	5.20	0.69	
KTW-1 month	i-PRF	4.07	2.61	5.80	0.75	0.964
	HA	4.09	2.83	5.21	0.69	
KTW-3 months	i-PRF	4.08	2.61	5.80	0.75	0.979
	HA	4.09	2.84	5.86	0.71	

**Table 6:** Pairwise comparisons of changes in the gingival index between assessment times in each group i-PRF: injectable platelet-rich fibrin; HA: hyaluronic acid *Wilcoxon test was used for the intragroup comparison (when the same variable was checked across multiple time points in the intragroup comparisons).

Variables		Mean difference	95% CI of the difference (lower bound-upper bound)		P-value*
i-PRF group	Baseline-1 month	0.664	0.597	0.731	0.002
		0.542	0.430	0.655	
	Baseline-3 months	0.664	0.597	0.731	0.017
		0.571	0.471	0.671	
	1 month-3 months	0.542	0.430	0.655	0.384
		0.571	0.471	0.671	
HA group	Baseline-1 month	0.664	0.597	0.731	0.002
		0.542	0.430	0.655	
	Baseline-3 months	0.664	0.597	0.731	0.017
		0.571	0.471	0.671	
	1 month-3 months	0.542	0.430	0.655	0.384
		0.571	0.471	0.671	

**Table 7:** Comparison of the gingival index between the two groups at the three assessment times (n=42) GI: gingival index; i-PRF: injectable platelet-rich fibrin; HA: hyaluronic acid *Mann-Whitney U test was used for the intergroup comparisons (i-PRF versus HA).

	Groups	Mean	Minimum value	Maximum value	Standard deviation	P-value*
GI-baseline	i-PRF	0.66	0.40	1.00	0.22	1.000
	HA	0.66	0.40	1.00	0.22	
GI-1 month	i-PRF	0.54	0.00	1.00	0.36	1.000
	HA	0.54	0.00	1.00	0.36	
GI-3 months	i-PRF	0.57	0.10	1.00	0.32	1.000
	HA	0.57	0.10	1.00	0.32	

**Table 8:** Pairwise comparisons of changes in the probing depth between assessment times in each group i-PRF: injectable platelet-rich fibrin; HA: hyaluronic acid *Wilcoxon test was used for the intragroup comparison (when the same variable was checked across multiple time points in the intragroup comparisons).

Variables		Mean difference	95% CI of the difference (lower bound-upper bound)		P-value*
i-PRF group	Baseline-1 month	1.33	1.18	1.48	1.000
		1.33	1.18	1.48	
	Baseline-3 months	1.33	1.18	1.48	0.973
		1.33	1.15	1.51	
	1 month-3 months	1.33	1.18	1.48	0.957
		1.33	1.15	1.51	
HA group	Baseline-1 month	1.42	1.27	1.58	0.251
		1.30	1.13	1.48	
	Baseline-3 months	1.42	1.27	1.58	0.539
		1.35	1.17	1.53	
	1 month-3 months	1.30	1.13	1.48	0.723
		1.35	1.17	1.53	

**Table 9:** Comparison of the probing depth between the two groups at the three assessment times (n=42) PD: probing depth; i-PRF: injectable platelet-rich fibrin; HA: hyaluronic acid *Mann-Whitney U test was used for the intergroup comparisons (i-PRF versus HA).

	Groups	Mean	Minimum value	Maximum value	Standard deviation	P-value*
PD-baseline	i-PRF	1.33	1.00	2.00	0.48	0.372
	HA	1.43	1.00	2.00	0.50	
PD-1 month	i-PRF	1.33	1.00	2.00	0.48	0.582
	HA	1.31	1.00	3.00	0.56	
PD-3 months	i-PRF	1.33	1.00	3.00	0.57	0.823
	HA	1.36	1.00	3.00	0.58	

**Table 10:** Pairwise comparisons of changes in the bleeding on probing between assessments times in each group i-PRF: injectable platelet-rich fibrin; HA: hyaluronic acid *Wilcoxon test was used for the intragroup comparisons (when the same variable was checked across multiple time points in the intragroup comparisons).

Variables		Mean difference	95% CI of the difference (lower bound-upper bound)		P-value*
i-PRF group	Baseline-1 month	0.238	0.103	0.372	0.3170
		0.333	0.184	0.482	
	Baseline-3 months	0.238	0.103	0.372	0.157
		0.381	0.227	0.534	
	1 month-3 months	0.333	0.184	0.482	0.593
		0.381	0.227	0.534	
HA group	Baseline-1 month	0.333	0.184	0.482	1.000
		0.333	0.184	0.482	
	Baseline-3 months	0.333	0.184	0.482	0.617
		0.381	0.227	0.534	
	1 month-3 months	0.333	0.184	0.482	0.637
		0.381	0.227	0.534	

**Table 11:** Comparison of the bleeding on probing index between the two groups at the three assessment times (n=42) BOP: bleeding on probing; i-PRF: injectable platelet-rich fibrin; HA: hyaluronic acid *Mann-Whitney U test was used for the intergroup comparisons (i-PRF versus HA).

	Groups	Mean	Minimum value	Maximum value	Standard deviation	P-value*
BOP-baseline	i-PRF	0.24	0.00	1.00	0.43	0.337
	HA	0.33	0.00	1.00	0.48	
BOP-1 month	i-PRF	0.33	0.00	1.00	0.48	1.000
	HA	0.33	0.00	1.00	0.48	
BOP-3 months	i-PRF	0.38	0.00	1.00	0.49	1.000
	HA	0.38	0.00	1.00	0.49	

## Discussion

A thick gingival phenotype has been considered more favorable than a thin gingival phenotype in distinct clinical procedures [26]. It is widely known that sites displaying a thin gingival phenotype, in addition to a lack of KTW, are more prone to the occurrence of gingival recession [27]. Thick tissue phenotype has been associated with better outcomes following corrective periodontal procedures, such as root coverage [28]. With a CAF, a thicker flap, i.e., the GT greater than 0.8 mm, resulted in better root coverage when compared to flaps with a thin GT, i.e., less than 0.8 mm [7]. Less post-treatment recession was reported after guided tissue regeneration procedures with tissue thickness >1 mm compared to sites where GT is less than 1 mm [29]. A thicker biotype has been correlated with greater tissue rebound following surgical crown lengthening [30]. Greater mean bone loss was observed around implants in sites with thin as compared to thick overlying mucosa [31].

Even in orthodontics, a higher incidence of gingival recession has been reported in teeth exhibiting a thin periodontal phenotype exposed to orthodontic forces intended to move the dentition buccally [32]. Thus, modifying the gingival phenotype from thin to thick predicts “a more favorable environment for preventing disease and maintaining periodontal health” [28]. In literature, distinct surgical approaches have been used to modify the gingival phenotype such as autologous gingival grafts, acellular dermal matrix, PRF membranes, and recently, fetal membrane [27,33-35]. Two non-surgical minimally invasive methods have been studied in our study for this purpose. Ozsagir et al. and Fotani et al. suggested that multiple sessions of i-PRF injections in thin gingiva resulted in an increased GT and KTW, as i-PRF is rich in high physiological amounts of regenerative cells and growth factors [36-37].

In the current study, injections of cross-linked HA were proposed for the purpose of enhancing the gingival phenotype, as HA could accelerate the proliferation of gingival fibroblasts, promote the formation of collagen, and eventually induce soft-tissue augmentation [38]. The slow degradation pattern of cross-linked HA prolongs its presence throughout the various phases of wound healing, thus promoting healing by regeneration instead of reparation [39]. We used the transgingival probing method for the quantitative measurement of GT, as this method is considered reliable, and reproducible.

GT and KTW were the main outcomes in our present study. A significant increase in GT and KTW was observed in the HA group after 1 month and 3 months compared to baseline in the follow-up period. In agreement with the present results, a recent study conducted on dogs by Shirakata et al. in 2021 concluded that HA in conjunction with CAF enhances periodontal regeneration and wound healing in gingival recession defects. The CAF/HA group showed a statistically significant reduction in the width of gingival recession (p < 0.01) and a significantly higher formation of connective tissue attachment in the CAF/HA group compared with the CAF group [40].

Various studies used different concentrations of HA for the purpose of papilla regeneration, this can be explained by the property of HA to induce neovascularization. In a recent RCT evaluating the effect of cross-linked HA, deficient papillae were injected with HA on one side of the anterior maxilla, while physiological saline was injected into papillae on the other side. A significant increase in papillae injected with HA was reported after 3 and 6 months. The in-vitro study also revealed that HA also significantly accelerated the proliferation and migration of gingival fibroblasts. In 2017, Pi et al. investigated the effect of HA and phosphate buffer solution (PBS) when injected into interdental papillae in female rats. HA group showed a significant increase in papillary volume when compared to the PBS group. They reported that new micro-vascularity was observed in the connective tissue layer of the interdental papillae injected with HA fillers with no inflammatory infiltrates formed in rats [41].

A study by Wang et al. 2007 concluded that cross-linked HA stimulates collagen synthesis when injected into the photodamaged skin of a forearm. The study believed that the mechanical stretching of the dermis caused by HA leads to stretching and activation of dermal fibroblasts and ultimately, induces collagen production [42]. Another study by Prato et al. evaluated the use of an autologous-cell HA graft for gingival augmentation in mucogingival surgery. In six patients requiring keratinized tissue augmentation, autologous human fibroblasts were obtained from the gingivae and cultured on a non-woven matrix of the benzyl ester of HA. The graft was adapted and sutured over the exposed periosteum. The authors reported an increased amount of fully keratinized tissue after 3 months with an average KT width gain of 2 ± 0.4 mm [43]. In the i-PRF group, a statistically significant increase in GT and KTW was also noted between baseline-1 month and baseline-3 months.

These outcomes are in agreement with a previous study by Ozsagir et al. who randomly treated patients with thin gingiva [19]. They injected i-PRF on one side with a 30-gauge needle while on the other side with a 24-gauge needle and reported an increase in GT within both groups and a statistically significant increase in KTW in the 30-gauge needle group. Another study by Ozsagir et al. in 2020 compared micro-needling (MN) with i-PRF and i-PRF alone to enhance the gingival phenotype [36]. I-PRF was injected on one side, and MN+ i-PRF was performed on the opposite side of the same patient. This procedure was repeated for four sessions with 10 days between sessions. GT significantly increased after 3 and 6 months in both groups, while KTW significantly increased in the MN+ i-PRF group only [36].

Fotani et al. also reported a statistically significant increase in the GT and KTW at 1 month and 3 months after injecting i-PRF into the gingival sulcus of individuals with thin gingival biotype [37]. A statistically significant decrease after 1 and 3 months was noted in both HA and i-PRF groups for the GI, and no statistically significant differences were observed in BOP and PD after 1 and 3 months, suggesting good oral hygiene by the patient and that both methods do not cause an inflammatory response.

Strengths and limitations

Our study was the first to evaluate injecting HA for the purpose of increasing the GT and KTW and modifying the gingival phenotype. The study also compared HA with i-PRF, a new minimally invasive non-surgical method that successfully increased GT and KTW. However, the short follow-up period and relatively small sample size were among the limitations of the current work.

## Conclusions

Multiple injections of HA and i-PRF in thin gingival phenotypes resulted in an increased GT and KTW with no statistically significant differences between the two methods. Both minimally invasive methods were more effective in increasing the gingival width than the KTW. Further studies with longer follow-up periods are required to confirm the current findings.
